# Dramatic evolution of body length due to postembryonic changes in cell size in a newly discovered close relative of *Caenorhabditis elegans*

**DOI:** 10.1002/evl3.67

**Published:** 2018-07-16

**Authors:** Gavin C. Woodruff, John H. Willis, Patrick C. Phillips

**Affiliations:** ^1^ Forestry and Forest Products Research Institute Forest Pathology Laboratory Tsukuba Japan; ^2^ Department of Biology, Institute of Ecology and Evolution University of Oregon Eugene Oregon 97403

**Keywords:** Body size, developmental constraint, evo‐devo, *C. elegans*, macroevolution

## Abstract

Understanding morphological diversity—and morphological constraint—has been a central question in evolutionary biology since its inception. Nematodes of the genus *Caenorhabditis*, which contains the well‐studied model organism *C. elegans*, display remarkable morphological consistency in the face of extensive genetic divergence. Here, we provide a description of the broad developmental patterns of a newly discovered species, *C*. sp. 34, which was isolated from fresh figs in Okinawa and which is among the closest known relatives of *C. elegans*. *C*. sp. 34 displays an extremely large body size; it can grow to be nearly twice as long as *C. elegans* and all other known members of the genus. Observations of the timing of developmental milestones reveal that *C*. sp. 34 develops about twice as slowly as *C. elegans*. Measurements of embryonic and larval size show that the size difference between *C*. sp. 34 and *C. elegans* is largely due to postembryonic events, particularly during the transition from larval to adult stages. This difference in size is not attributable to differences in germ line chromosome number or the number of somatic cells. The overall difference in body size is therefore largely attributable to changes in cell size via increased cytoplasmic volume. Because of its close relationship to *C. elegans*, the distinctness of *C*. sp. 34 provides an ideal system for the detailed analysis of evolutionary diversification. The context of over 40 years of *C. elegans* developmental genetics also reveals clues into how natural selection and developmental constraint act jointly to promote patterns of morphological stasis and divergence in this group.

**Impact Summary**

Despite the ability of artificial selection to change nearly any phenotypic trait we can think of, the predominant observation in nature is the persistence of phenotypic stasis over geological timescales. Multiple explanations for this pattern have been proposed, most notably stabilizing selection and developmental constraint. Yet despite years of debate, the paradox of stasis remains yet to be fully resolved. We address this by for the first time describing the developmental basis of very large body size in *C*. sp. 34, a new species associated with fresh *Ficus* figs that happens to be the closest known relative *C*. *elegans*. Surprisingly, *C*. sp. 34 adults are nearly twice as long as *C. elegans* adults, whereas *C*. sp. 34 embryos are only about 20% longer than *C. elegans* embryos. Furthermore, we show that the number of cells is unchanged in this species despite its large size, revealing a largely conserved cell lineage underlies extreme morphological divergence. In concert with what is known about body size mutants in *C. elegans*, we conclude that selection and developmental constraint act jointly to promote the pattern of body plans observed in *Caenorhabditis*.

It is natural for evolutionary biologists to focus on change; the more dramatic, the better. However, we expect species to accumulate substantial differences from one another over time even in the absence of natural selection (Lynch 1990). In fact, even across fairly diverse groups, the predominant pattern of evolution is one of constrained variation in morphological diversity rather than diversification per se (Hansen and Houle 2004). For the last 40 years, the biological bases of limits to macroevolutionary variation have been hotly debated (Eldredge and Gould 1972; Charlesworth *et al*. 1982; Smith *et al*. 1985; Futuyma 2010). In the early phases of this discussion, evolutionary geneticists tended to argue that long‐term limits to variation must be generated by stabilizing selection in which the natural tendency for species to move apart from one another in morphological space due to the accumulation of new mutations via genetic drift is strongly counterbalanced by natural selection against individuals that do not adhere to an “optimal” phenotype (Charlesworth *et al*. 1982). In contrast, evolutionary developmental biologists and paleontologists often argued that development systems themselves constrain the actual production of variation that is the basis of evolutionary change, such that species that share common developmental regulatory systems would be expected to show limited phenotypic differences from one another (Smith *et al*. 1985). In the intervening years, it has become clear that the actual diversity that we observe in nature must somehow be a balance between these different sources of constraint (Hansen and Houle 2004; Futuyma 2010).

Nematodes are a particularly compelling example of extremely constrained morphological evolution. For instance, species within the genus *Caenorhabditis*, which includes the important *C. elegans* model system, display such little morphological diversity that they are essentially impossible to tell apart except in a few finer details of male tail morphology (Kiontke *et al*. 2011; Félix *et al*. 2014). Indeed, species are defined in part via their ability to cross with one another (Félix *et al*. 2014). Yet this morphological conservatism is in stark contrast to amount of diversity observed at the level of DNA sequence. Here, different species within this group are as divergent from one another as mice are from lampreys (Kiontke *et al*. 2004). Nematodes are famous for having a very stereotypical pattern of development, with a largely fixed lineage of cell division events and number of adult somatic cells (Sternberg and Horvitz 1982; Sulston *et al*. 1983; Delattre and Félix 2001; Zhao *et al*. 2008; Schulze and Schierenberg 2011). Is the constrained pattern of morphological diversity observed within this genus generated by development or selection? Here, we test this hypothesis using the developmental characteristics of a recently discovered relative of *C. elegans*, *C*. sp. 34 (Kanzaki *et al*. 2018). In addition to exhibiting exceptional differences in body size and other morphological characteristics, this species is also distinctive from other *Caenorhabditis* species in its developmental rate and ecological niche. We describe a number of these features, with a particular focus on examining the proximal causes of the extreme difference in body size.

## Methods

### STRAINS

**Figure 1:**
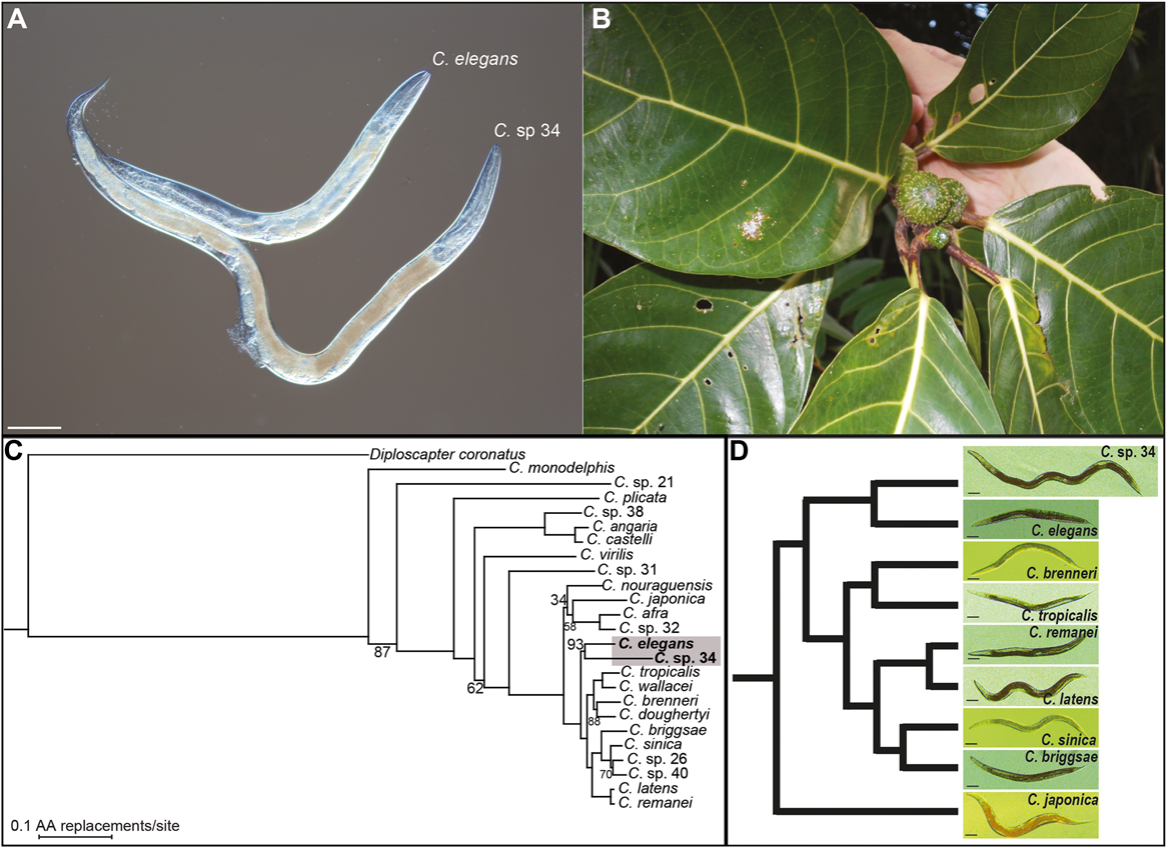
*C*. sp. 34 is a morphologically and ecologically distinct species of *Caenorhabditis*. (A) *C*. sp. 34 is longer than *C. elegans*. (B) *C*. sp. 34 is associated with the fresh, intact figs of *Ficus septica*, in contrast to most *Caenorhabditis* species, which are associated with rotting plant material (Kiontke *et al*. 2011). (C) A maximum likelihood phylogenetic analysis of 24 *Caenorhabditis* species suggests *C*. sp. 34 is a close relative of *C. elegans*. Measures of node support are out of 100 bootstrap replicates, and unlabeled nodes were recovered in all bootstrap replicates. The scale bar represents 0.1 amino acid replacements/site. The *C*. sp. 34‐*C. elegans* clade was recovered in 93/100 bootstrap replicates (Document S1). The topology of this tree is largely consistent with previous studies (but see Document S1) (Kiontke *et al*. 2011; Slos *et al*. 2017). Species names as in (Félix *et al*. 2014; Huang *et al*. 2014; Slos *et al*. 2017). (D) *C*. sp. 34 is a morphologically exceptional *Caenorhabditis*. Age‐synchronized *Caenorhabditis* females/hermaphrodites across nine species shows *C*. sp. 34 to be highly derived in its body length. L4 larvae raised at 25°C were isolated and imaged 2 (*C*. sp. 34) or 1 (all other species) days later; older *C*. sp. 34 animals were used to account for differences in developmental timing (Fig. 3). The cladogram follows the analysis in (C). All scale bars are 100 microns.

*C*. sp. 34 was originally isolated from a fresh fig of the tree *Ficus septica* in May 2013 on Ishigaki Island, Okinawa Prefecture, Japan (Latitude 24°24’38.06” N, Longitude 124°11’06.81” E) (Kanzaki *et al*. 2018). The fig was dissected in M9 buffer, and live worms in buffer were transferred to a culture plate. The nonisofemale lines NKZ1 and NKZ2 were derived from the same population (also referred to as strain NK74SC), and they are the result of two replicates of an attempt to remove microbial contaminants that have been maintained separately in culture since 2014. *C*. *elegans* strains N2 and JK574 *fog‐2 (q71)*(Schedl and Kimble 1988) were used for most comparisons. Live females/hermaphrodites of *C. briggsae* AF16, *C. remanei* EM464, *C*. *latens* VX88, *C*. *tropicalis* JU1373, *C*. *sinica* JU727, *C. japonica* DF5081, and *C. brenneri* CB5161 were used to illustrate the general morphological constancy of the genus in Figure 1. Animals were maintained on Nematode Growth Media (with 3.2% agar to discourage burrowing) supplemented with *Escherichia coli* strain OP50‐1 for food.

### DNA PREPARATION, AMPLIFICATION, SEQUENCING, AND DE NOVO ASSEMBLY

Twenty‐one individual, ethanol‐fixed *C*. sp. 34 specimens were utilized as a source for sequence data. Individuals were collected from dissected fresh *F. septica* figs from the Okinawan islands of Ishigaki, Iriomote, and Yonaguni in May 2016 (Fig. S1, Table S1). Live individuals were immediately fixed in 100% ethanol and kept at –20°C for 3–11 months. Individual animals were then washed three times in PBS, transferred to individual tubes, and then digested with Proteinase K in PBS‐EDTA in 20 μl reactions. After Proteinase K inactivation, 10 μl of these reactions then underwent linear amplification with the Illustra GenomiPhi V3 amplification kit (GE Lifesciences). DNA was subsequently purified with the Zymo DNA Clean and Concentrator kit.

EcoRI RAD libraries were prepared from amplified genomic DNA using a more recent RAD protocol that generates fewer PCR duplicates during library preparation (Ali *et al*. 2016). Paired‐end 150 bp reads were generated with the Illumina Hi‐Seq 4000. As the 3'‐end of resultant reads were of low quality, the last 50 bp of all reads were removed. Reads were then reoriented for Stacks with Flip2BeRAD (https://github.com/tylerhether/Flip2BeRAD), demultiplexed and quality filtered with the Stacks package (Catchen *et al*. 2013). Overlapping paired reads were merged with FLASH (Magoč and Salzberg 2011).

All processed *C*. sp. 34 reads from both sequencing runs were combined into a single fastq file, and Velvet (Zerbino and Birney 2008) was used to generate an incomplete de novo genome assembly using default parameters. Assembled contigs with ≥2x coverage were then utilized for phylogenetic analysis.

### PHYLOGENETIC ANALYSIS

Twenty‐four taxa (23 *Caenorhabditis* species: *C. afra*, *C. angaria*, *C. brenneri*, *C. briggsae*, *C. castelli*, *C. doughertyi*, *C. elegans*, *C. japonica*, *C. latens*, *C. monodelphis*, *C. nouraguensis*, *C. plicata*, *C. remanei*, *C. sinica*, *C*. sp. 21, *C*. sp. 26, *C*. sp. 31, *C*. sp. 32, *C*. sp. 38, *C*. sp. 40, *C*. *tropicalis*, *C. virilis*, and *C. wallacei*; and one outgroup, *Diploscapter coronatus* (Hiraki *et al*. 2017)) were used to identify orthologous protein‐coding loci for phylogenetic analysis. Protein sequences were retrieved from WormBase ParaSite (version WBPS9) (Howe *et al*. 2016) and the *Caenorhabditis* Genomes Project website (caenorhabditis.org; version CPG2) (Slos *et al*. 2017). Orthologous groups were obtained with OrthoFinder (Emms and Kelly 2015), but only four single‐copy orthologs were identified across all taxa. However, *D. coronatus* is known to have high heterozygosity and contains 8046 heterozygous gene pairs (or “allelic partners”) that are homologous to single‐copy genes in *C. elegans* (Hiraki *et al*. 2017). Taking this into account, OrthoFinder identified 457 orthologous groups if *D. coronatus* is allowed to have 1–2 copies of an ortholog, whereas all other *Caenorhabditis* taxa are allowed to only have one copy. After arbitrarily discarding one *D. coronatus* allelic partner for downstream analyses, these orthologous protein sequences were aligned with MAFFT(Katoh and Standley 2013) and trimmed with trimAL (Capella‐Gutiérrez *et al*. 2009).

To identify orthologous sequences in *C*. sp. 34, majority‐rule consensus sequences were generated from the previously described alignments using the *cons* function in the EMBOSS package. These consensus sequences were then aligned to the previously described *C*. sp. 34 de novo genome assembly with the *tblastn* function in BLAST+. Subsequent downstream analyses were performed in a manner similar to (Slos *et al*. 2017). After removing duplicate hits and sequences lacking spurious stop codons, 308 *C*. sp. 34 homologs were realigned to the previously retrieved *Caenorhabditis* and *D. coronatus* orthologs with MAFFT and again trimmed with trimAL (with option ‐gt 1 to remove all gaps). Alignments were then inspected to remove loci with spurious, clearly nonhomologous *C*. sp. 34 sequence. The remaining alignments from 287 protein‐coding loci were then concatenated with FASconCAT (Kück and Longo 2014) and used for phylogenetic analysis.

Phylogenetic relationships were inferred from the concatenated alignment (with 25 taxa, 287 protein‐coding loci (Table S2), 11,572 amino acids, and an average of 39 amino acids per locus) with RAxML (Stamatakis 2014) with the option PROTGAMMAAUTO to determine the protein substitution model. One Hundred bootstrap replicates were performed to ascertain confidence in the topology.

### SIZE AND MORPHOLOGICAL MEASUREMENTS

**Figure 2:**
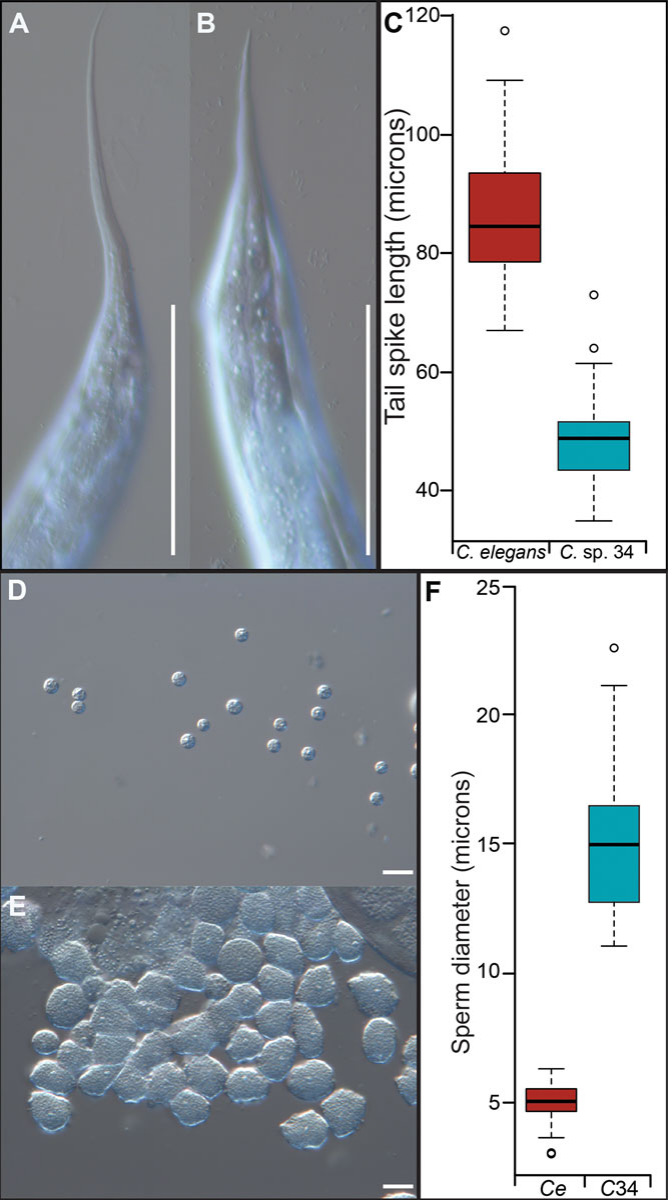
*C*. sp. 34 has small female tail spikes and giant sperm. *C*. *elegans* N2 hermaphrodite (A) and *C*. sp. 34 NKZ1 female (B) tail spikes. *C*. *elegans* hermaphrodite tail spikes had an average length of 86.5 microns (*N* = 43 worms, ± 5.3 SDM), whereas *C*. sp. 34 female tail spikes had an average length of 48.3 microns (Mann–Whitney U *P* < 0.0001, *N* = 41 worms, ± 3.7 SDM). Scale bars are 100 microns in both photos. (C) Quantification of tail spike length. *C*. *elegans n* = 43, *C*. sp. 34 *n* = 41. (D) Sperm dissected from *C*. *elegans (fog‐2)* males. (E) Sperm dissected from *C*. sp. 34 NKZ1 males. (F) Quantification of sperm size diameter. *C*. *elegans (fog‐2)* male sperm had an average diameter of 5.06 microns (*N* = 56 sperm, ± 0.36 SDM), whereas *C*. sp. 34 sperm had an average diameter of 15.07 microns (Mann–Whitney U *P* < 0.0001, *N* = 45 sperm, ± 1.37 SDM). Scale bars are 10 microns in both photos. For boxplots, the solid horizontal line is the median, the box represents the interquartile range, and the whiskers define the range excepting outliers (circles).

For comparing the growth of *C*. *elegans* N2 and *C*. sp. 34 NKZ1 over time (Fig. 5C), animals were synchronized by transferring early‐stage embryos (i.e., younger than the twofold stage) to new plates. Each day, a fraction of the synchronized animals was mounted on agar pads in 0.2 mM levamisole, imaged under Nomarski optics, and photographed. Animals were raised at 25°C. L4 *C*. sp. 34 females were moved to a new plate before adulthood to prevent mating and the confusion of the synchronized population with their progeny. *C*. *elegans* hermaphrodites were transferred to new plates every day after adulthood for the same purpose. Phenotypically diagnosable males were not used for length measurements. Images were analyzed with the ImageJ software (Schneider *et al*. 2012) to determine length measurements. Curved lines were also accounted for with the “segmented line tool” in ImageJ (see Fig. 2 in (Mörck and Pilon 2006)).

For comparing the sizes of *C*. *elegans* N2 and *C*. sp. 34 NKZ2 at comparable developmental stages (Fig. 5D–K), animals were synchronized by incubating mixed stage animals in a bleaching solution (1 part 10 M KOH: 6 parts sodium hypochlorite: 33 parts water) for seven (*C*. *elegans*) or 4.5 (*C*. sp. 34) minutes. Embryos were then washed four times in M9 buffer and allowed to hatch and arrest in the L1 larval stage overnight at room temperature. Larvae were transferred to bacteria‐seeded plates the next day and shifted to 25°C. Observations of developmental timing (described below) were used to determine the timing of the larval stages in *C. elegans* and *C*. sp. 34. Phenotypically diagnosable males were not used for length measurements. Animals at given larval stages were imaged with a dissecting microscope (at 10x magnification), photographed, and analyzed as above to determine length.

Female/hermaphrodites tail spikes, embryos, and sperm of *C*. *elegans* and *C*. sp. 34 were imaged under Nomarski microscopy (at 40x magnification) and analyzed with ImageJ to quantify morphological differences. Sperm of *C*. *elegans* (*fog‐2*) and *C*. sp. 34 NKZ1 males were isolated by cutting off male tails in M9 buffer with a needle.

### DEVELOPMENTAL TIMING

*C. elegans* N2 and *C*. sp. 34 NKZ2 animals were synchronized to the L1 stage as described above. Populations staggered 12 hours apart at 25°C were monitored hourly for the presence of actively molting individuals. Additionally, female vulva and male tail morphology was used to determine the fraction of L4 larvae and adults at a given time. *C*. *elegans* populations were monitored until all individuals developed into adults. *C*. sp. 34 populations were assayed for seven hours after the maximum adult fraction was attained.

### PLOIDY, NUCLEUS NUMBER, AND MORPHOMETRICS

Ploidy and nucleus observations were made using animals stained with the DNA‐staining Hoechst 33342 dye. *C. elegans fog‐2 (q71)* and *C*. sp. 34 NKZ2 young adult females were obtained by moving L4 females to new plates at 25°C and preparing them one (*C*. *elegans*) or two (*C*. sp. 34) days later for fluorescence microscopy. Animals were then fixed in 100% methanol for ten minutes at –20°C. Animals were washed three times in PBS and were then incubated in 1 μg/ml Hoechst 33342 for 10 minutes. Animals were washed three times in PBS and then mounted in 50% glycerol for visualization. Specimens for cell number, ploidy, and morphometrics were imaged with an Olympus FluoView 1000 laser‐scanning confocal microscope and its native Windows‐based FV10‐ASW software for image acquisition. A fraction of specimens was examined for germ line ploidy using a conventional compound microscope equipped with fluorescence.

For the determination of germ line ploidy, proximal oocytes in prophase I arrest were imaged and diakinesis chromosomes counted. For the determination of somatic nucleus number, z‐stacks with one micron steps across the whole specimen were generated. All somatic nuclei were then hand counted using the cell counter plugin in the ImageJ software (Schneider *et al*. 2012). ImageJ was then used to also determine the distance between homologous morphological landmarks using the same sets of images. Homologous landmarks were determined by anatomical similarity and the relative positions of nuclei. The morphological landmarks used were: the most anterior nucleus observed (likely Hyp4, number 1 on Fig. 6B); the most anterior intestinal nucleus (Int4, #2); the BDUL neuron (#3); the anterior spermatheca (measured at the end of the proximal ‐1 oocyte, #4); the center of the vulva (#5); the posterior spermatheca (measured at the end of the proximal ‐1 oocyte, #6); the VD11 neuron (#7); the most posterior intestinal nucleus (Int9, #8); and the most posterior nucleus (likely Hyp10, #9).

The images used for estimating the total number of somatic nuclei and the distances between homologous morphological landmarks were also used to measure nucleus size, somatic nuclear DNA content, and midbody hypodermal nucleus number. The segmented line tool and measure function in ImageJ were used to encircle neuronal, hypodermal, and intestinal nuclei to measure their area and pixel density. Fifteen worms per species were used, 15 hypodermal and neuronal nuclei per individual (and 7–15 intestinal nuclei per individual) were measured, and the average nuclear area per individual worm for each tissue type was used for subsequent analysis. The same nuclei used for area measurements were also used to estimate DNA content, which was done in a manner similar to previous reports (Flemming *et al*. 2000; Morita *et al*. 2002; Nyström *et al*. 2002; Lozano *et al*. 2006). Here, the average integrated density (defined as the “the product of area and mean gray value” in the ImageJ documentation (Schneider *et al*. 2012)) of each nucleus type per worm was measured. Assuming neuronal nuclei are diploid (2n), the per worm average hypodermal and intestinal nuclear integrated density measurements were divided by the half of the average neuronal nuclear integrated density to get an estimate of relative somatic nuclear DNA content in units of ploidy (or C‐value) (Lozano *et al*. 2006). Only ventral cord neurons were used for neuronal measurements, and only nuclei from the same image stack were used for measures of area and DNA content. Additionally, only hypodermal and neuronal nuclei posterior to the first intestinal ring and anterior to the vulva were used for measures of nuclear area and DNA content. For measures of midbody hypodermal nucleus number, all hypodermal nuclei between the most anterior nucleus of the first intestinal ring and the most posterior nucleus of the last intestinal ring were counted for each imaged worm. As this region is dominated by the large syncytial hypodermal cell Hyp7 (Altun *et al*. 2002–2006), this should account for most of the hypodermal nuclei in an individual.

## Results

### A MORPHOLOGICALLY NOVEL SPECIES OF FIG‐ASSOCIATED NEMATODE IS IN *CAENORHABDITIS*

**Figure 3:**
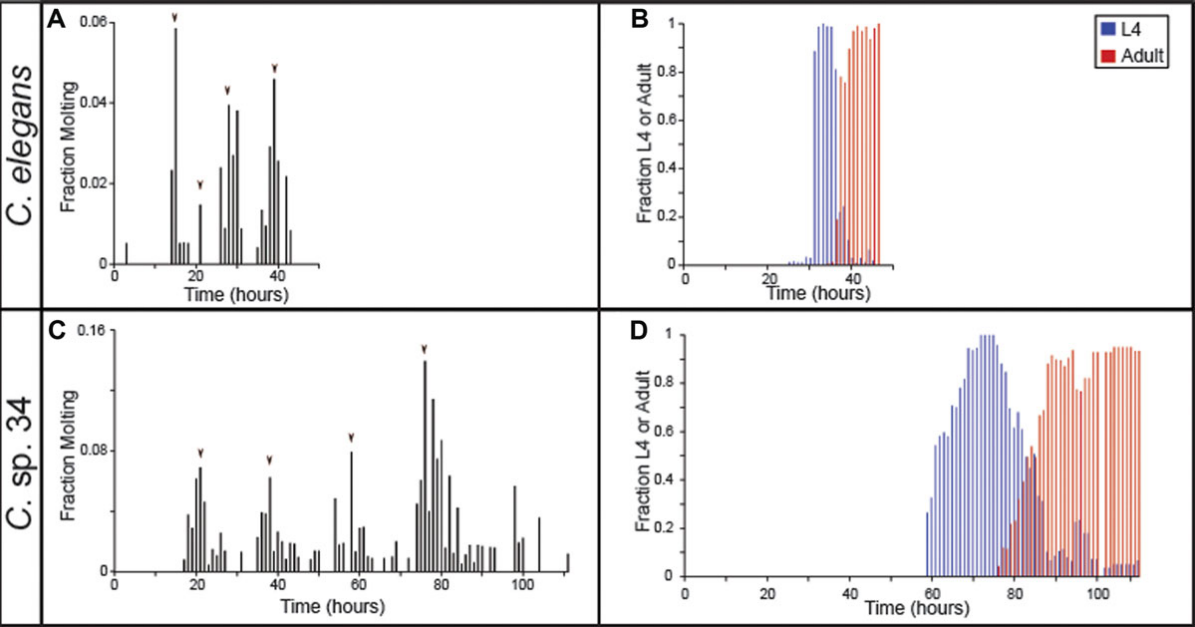
*C*. sp. 34 develops more slowly than *C. elegans*. Synchronized populations of *C. elegans* (A, B) and *C*. sp. 34 (C, D; for both species, average *N* worms = 107 ± 32.2 SDM; range = 23–445), were monitored hourly for the fraction of actively molting animals (A, C) and the fraction of L4 larvae and adults (B, D). Populations were synchronized at the L1 larval stage 12 hours apart and monitored concurrently to capture the full progression of development (i.e., animals were not monitored for 24 hours a day). Arrowheads in (A) and (C) represent the maximal molting fractions corresponding to the likely major molting events.

*C*. sp. 34 was originally isolated from the fresh, intact figs of *Ficus septica* in Okinawa, Japan (Kanzaki *et al*. 2018), and a subsequent phylogenetic analysis with 287 protein‐coding loci places this species among the closest reported relatives of *C. elegans* (Fig. 1; Document S1; see methods). *C*. sp. 34 is an exceptional *Caenorhabditis* species in a number of respects. In contrast to other morphologically indistinguishable species of the *Elegans* group, they are huge in size, on average 64% longer than its close relative *C. elegans* (Fig. 1A; Fig. 5, see below). *C*. sp. 34 females have a distinctive, stumpy tail morphology, with a much shorter tail spike than those of *C. elegans* hermaphrodites (Fig. 2A–C). In addition, *C*. sp. 34 has enormous sperm that are on average three times longer in diameter than those of *C. elegans* (Fig. 2D–F). *C*. sp. 34 also develops very slowly, with a generation time nearly twice as long as *C. elegans* (Fig. 3, see below). Mating tests between *C. elegans* and *C*. sp. 34 yielded no viable progeny (Document S2). *C*. sp. 34 is also exceptional in its ecological niche, with proliferating animals being found in fresh figs (Fig. 1B), whereas most *Caenorhabditis* animals are associated with rotting plant material (Kiontke *et al*. 2011).

### *C*. SP. 34 DEVELOPS SLOWLY

*C. elegans* typically takes about two days to develop at 25°C. However, it was readily apparent that *C*. sp. 34 has a much slower developmental rate. This was quantified by examining the fraction of animals actively molting and the number of animals in the L4 and adult stages (which can be easily ascertained morphologically) over time (Fig. 3). The four larval molts are highly conserved across nematodes (Sommer and Streit 2011), and this is reflected in the periodicity of the molting fraction of both *C. elegans* and *C*. sp. 34 (Fig. 3A and C). *C. elegans* had maximal molting fractions at 15, 21, 28, and 39 hours past L1 synchronization (Fig. 3A). Conversely, *C*. sp. 34 had maximal molting fractions at 21, 38, 58, and 76 hours (Fig. 3C), revealing a developmental rate that is about twice as slow. This difference is also apparent in the proportion of L4‐ and adult‐like animals over time. The maximal L4 and adult fractions occur in *C. elegans* at 34 and 46 hours past L1 synchronization, whereas in *C*. sp. 34 they are at 72 and 106 hours (Fig. 3B and D). In addition, there is much more variation in developmental rate in *C*. sp. 34 than *C. elegans*. The amount of time in which L4 larvae were observed was over twice as long in *C*. sp. 34 (53 hours) than in *C. elegans* (21 hours).

### *C*. SP. 34 IS NOT POLYPLOID

**Figure 4:**
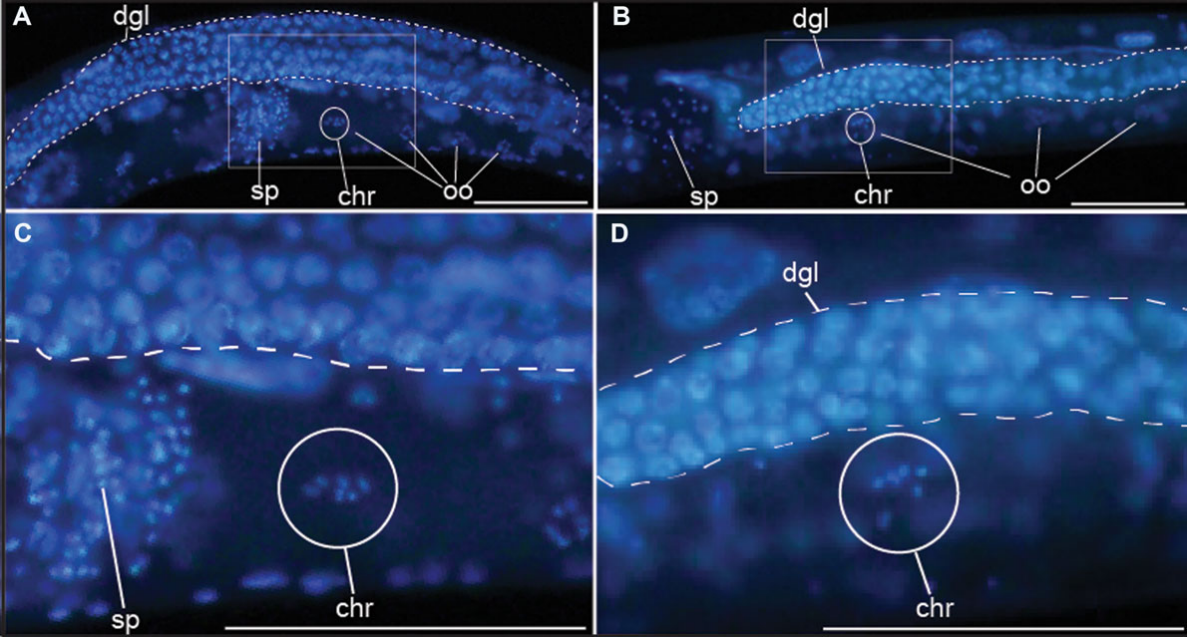
*C*. sp. 34 and *C. elegans* have the same number of chromosomes. DNA stained *C. elegans* (A, C) and *C*. sp. 34 (B, D) reveal that late‐prophase I oocytes contain six chromosomes (chr, encircled). Also of note is the reduced *C*. sp. 34 gonad relative to *C. elegans* (Fig. S3). Scale bars represent 100 microns in all photos. dgl, distal germ line. sp, sperm. oo, oocyte.

One explanation then for the increased size of *C*. sp. 34 is of a chromosome or genome duplication event. For instance, polyploid strains of *C. elegans* were initially generated to show that the X chromosome:autosome ratio was the major determinant of sex (Nigon and Félix 2017), but it was also noted that polyploid animals are larger than wild‐type (Lozano *et al*. 2006; Nigon and Félix 2017). Ploidy can be easily ascertained by examining DNA‐stained oocytes, which in *Caernorhabditis* arrest in prophase I prior to maturation (Greenstein 2005), allowing for chromosomes to be easily visualized (Fig. 4). In all *C*. sp. 34 specimens where the chromosomes in diakinesis‐stage oocytes were apparent (*n* = 29), six chromosomes were observed. This was also true of *C*. *elegans* (*n* = 15). In many *C*. sp. 34 animals, germ line and oocyte nuclear abnormalities were observed (Fig. S2). This may reflect oocyte endoreplication or chromosome condensation in the unmated animals used for microscopy, which can also be observed in older, sperm‐depleted *C*. *elegans* hermaphrodites (Detwiler *et al*. 2001). This may also reflect nutritional deficiencies in standard *C. elegans* laboratory confidtions for *C*. sp. 34, as starvation conditions are known to affect the germline in *C*. *elegans* (Angelo and Van Gilst 2009).

### *C*. SP. 34 LENGTH DIFFERENCE IS LARGELY DUE TO POSTEMBRYONIC EVENTS

**Figure 5:**
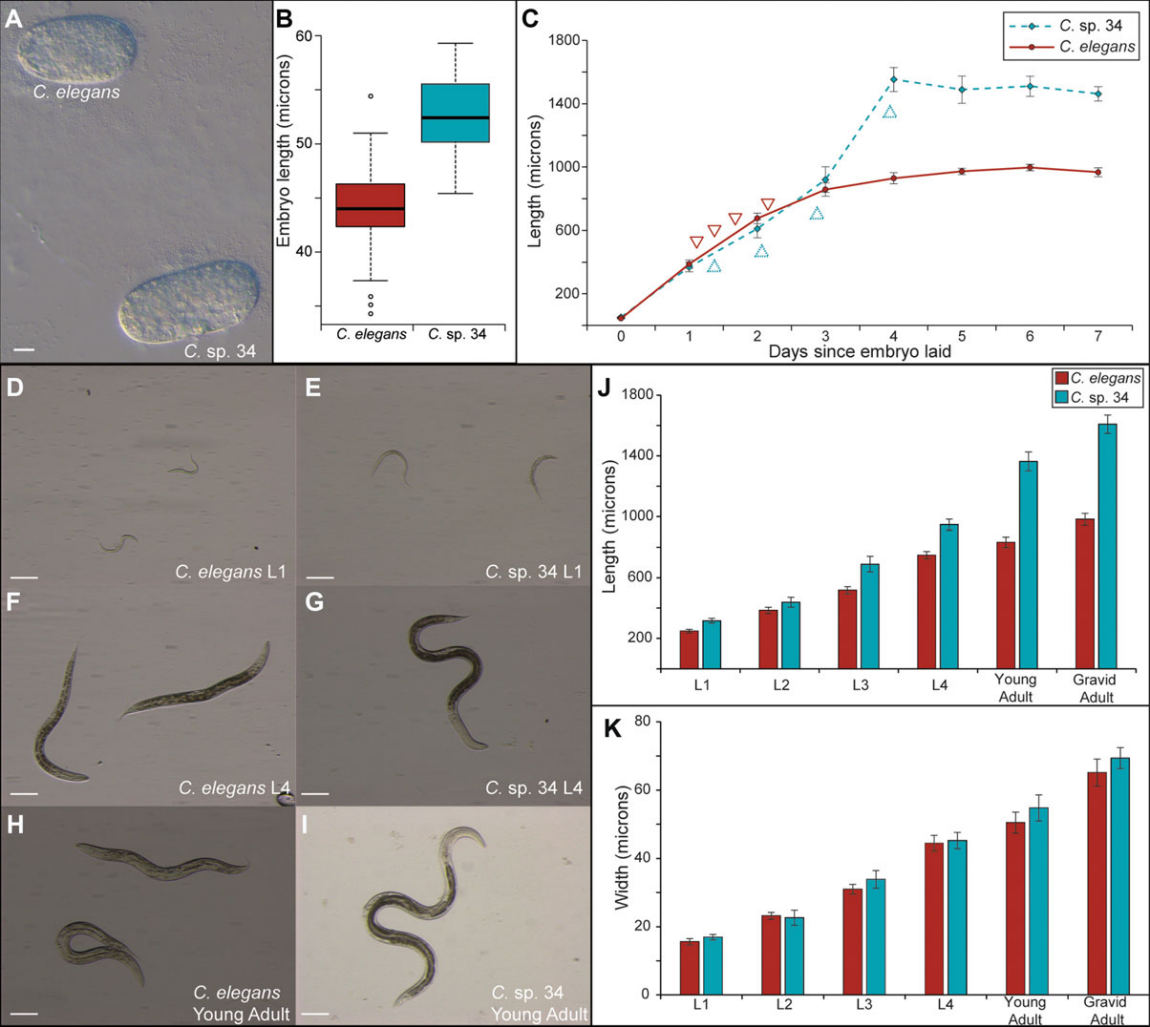
The length difference between *C. elegans* and *C*. sp. 34 is largely due to postembryonic events. (A) *C. elegans* and *C*. sp. 34 embryos. Scale bar = 10 microns. (B) Boxplot comparing embryo length (*n* = 61 for *C*. *elegans*; *n* = 35 for *C*. sp. 34; Mann–Whitney U *P* < 0.0001). *C*. sp. 34 embryos are on average 19% longer than *C*. *elegans* embryos. (C) Comparison of body length size over time in populations of *C*. sp. 34 and *C. elegans* synchronized as embryos (average *N* worms = 21 ± 3.7 SDM; range = 11–36). Data at time “0” is the same as in (B). Arrows correspond to estimates of larval molts in *C. elegans* (pointing down) and *C*. sp. 34 (pointing up) as determined in a Fig. 3. (D–I) Images of *C*. *elegans* (D, F, H) d *C*. sp. 34 (E, G, I) at developmentally comparable stages. Scale bars correspond to 100 microns in all panels. (J) Comparison of body length at developmental stages. *C*. sp. 34 is significantly longer than *C*. *elegans* at all stages (Mann–Whitney U *P* < 0.003 for all stages), but a 27% length difference at the L1 stage grows to a 64% difference in adults (Average *N* worms = 33 ± 4.1 SDM; range = 16–41). (K) Comparison of body width of same animals as in (J). The width of *C*. sp. 34 is comparable to *C*. *elegans* at all developmental stages. Error bars represent one standard deviation of the mean in panels (C, J–K).

To investigate the developmental basis of the size difference between *C*. sp. 34 and *C*. *elegans*, length was measured over time and developmental stage (Fig. 5). Despite being 64% longer on average than *C*. *elegans* at four days after egg‐laying, *C*. sp. 34 embryos are are only 19% longer than *C. elegans* embryos (Fig. 5A–C). Thus, it appears that a substantial portion of the length difference between these species is due to postembryonic events. However, as development is delayed in *C*. sp. 34 compared to *C*. *elegans* (Fig. 3), length comparisons at similar timepoints are problematic. To address this, the lengths of animals were compared at similar developmental stages (Fig. 5D–J). Although *C*. sp. 34 is longer than *C*. *elegans* at all developmental stages (Fig. 5J), between the L3 and adult stages the average length difference grows from 33% to 64%. Thus, much of the difference in length between species is developmentally regulated during the larva‐to‐adult transition. In addition, although *C*. sp. 34 adults are observed to be significantly wider than *C. elegans* (Mann–Whitney U *P* = 0.02), they are nominally wider on average by only four microns (Fig. 5K). The size difference between these species is thus dominated by length.

### *C*. SP. 34 SIZE DIFFERENCE IS DUE TO DIFFERENCES IN CELL SIZE AND NOT CELL NUMBER

**Figure 6:**
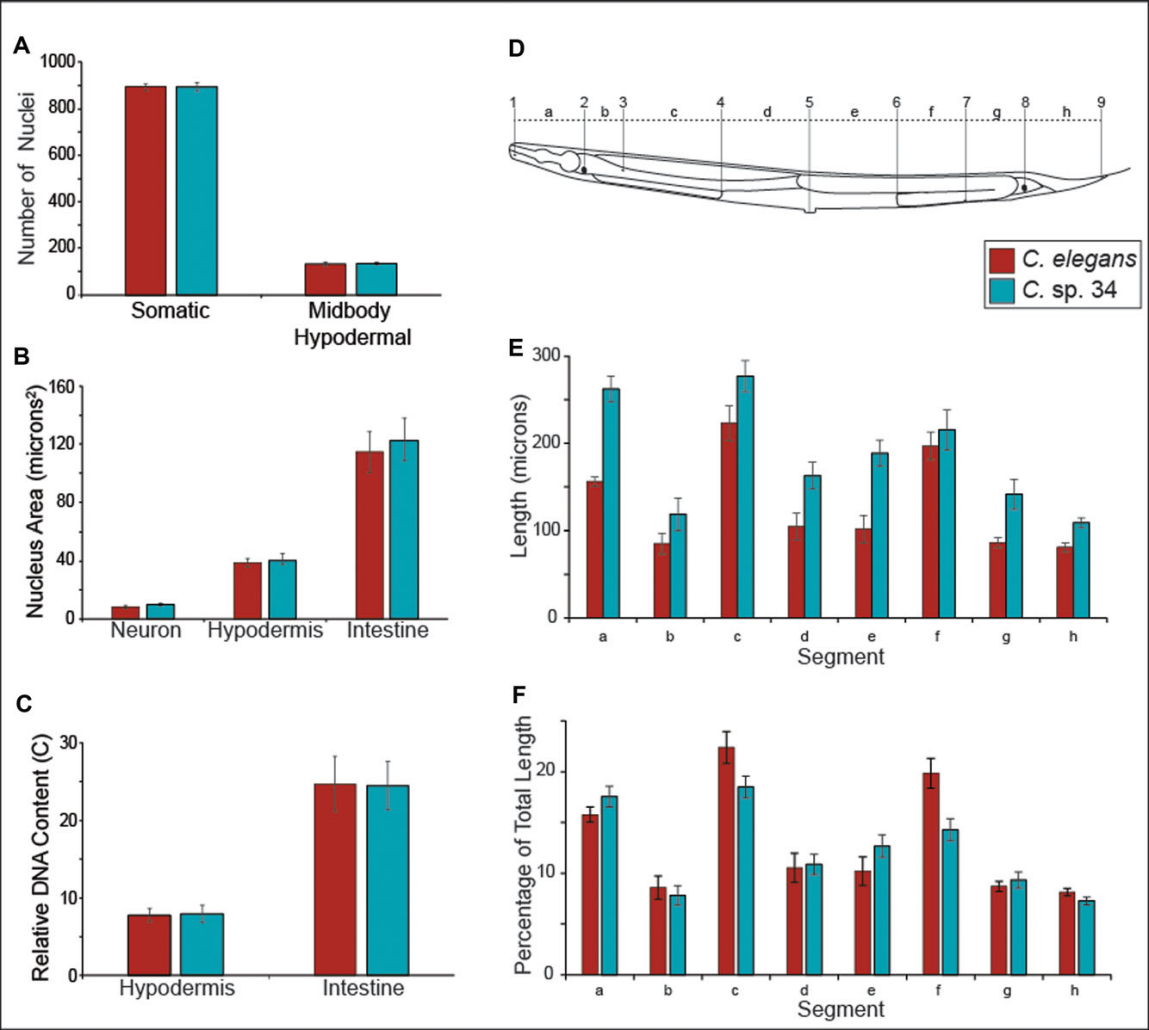
The size difference between *C. elegans* and *C*. sp. 34 is largely due to differences in cell size and not cell number. (A) Total number of somatic nuclei and midbody hypodermal nuclei (see methods) in young adult unmated *C*. sp. 34 and *C*. *elegans* (*fog‐2)* females. *fog‐2* animals make no sperm nor self‐progeny but are somatically identical to wild‐type hermaphrodites. No significant difference between somatic nucleus number (Mann–Whitney U *P* = 0.59) nor hypodermal nucleus number (Mann–Whitney U *P* = 0.29) was observed. *n* = 15 for both species. (B) Nuclear areas of neuronal, hypodermal, and intestinal nuclei in *C. elegans* and *C*. sp. 34. Data are derived from the same images used in (A). *n* = 15 worms for both species. No significant differences in nucleus area were detected for any cell type (neuron: Mann–Whitney U *P* = 0.12; hypodermis: Mann–Whitney U *P* = 0.46; intestine: Mann–Whitney U *P* = 0.54). (C) Estimates of DNA content of polyploid somatic tissues in *C. elegans* and *C*. sp. 34. Data are derived from the same images used in (A) and (B). No significant differences in somatic nuclear DNA content (see methods) were detected for either cell type (hypodermis: Mann–Whitney U *P* = 0.74; intestine: Mann–Whitney U *P* = 1). (D) Schematic of morphological markers used to measure length segments (adapted from WormAtlas(Altun *et al*. 2002–2006)). The distances (letters) between homologous nuclei (numbers) were compared between young adult *C*. sp. 34 and *C*. *elegans* (*fog‐2)* females. The specific morphological landmarks used are detailed in the experimental procedures. (E) The length between homologous morphological markers in *C*. *elegans* and *C*. sp. 34. All homologous segments are significantly longer in *C*. sp. 34, with the exception of segment f. *n* = 15 for *C. elegans*, *n* = 16 for *C*. sp. 34. (F) The percentage of the total body length of given homologous segments in *C*. sp. 34 and *C*. *elegans*. Same data as in (E). Although largely comparable in proportion, segments A, C, E, F, and H consist of a significantly different percentage of the total body size between *C*. *elegans* and *C*. sp. 34 (Mann–Whitney U *P*‐values for segments A–H: 0.009 (segment a); 0.29 (segment b); 0.00031 (segment c); 0.89 (segment d); 0.0063 (segment e); <0.0001 (segment f); 0.36 (segment g); 0.0036 (segment h). Error bars represent one standard deviation of the mean in panels (A–C, E and F).

All differences in body size must be due to differences in cell number, cell size, or both. To distinguish among these possibilities, somatic nucleus numbers (used as a proxy for cell number) were hand‐counted in unmated *C*. sp. 34 adult females and *C. elegans* (*fog‐2*) adult pseudo‐females. The *fog‐2* mutation was used to provide comparable specimens that lacked self‐embryos, which had the potential to contribute to error in somatic nucleus number estimation. In addition, *fog‐2* mutants have no known somatic defects (Schedl and Kimble 1988). Germ cells were not counted as *C*. sp. 34 germ lines are reduced relative to *C*. *elegans* (Fig. 4; Fig. S2; Fig. S3), and it is unlikely that this tissue would contribute to the length difference. No significant difference in somatic nuclei number between *C*. *elegans* and *C*. sp. 34 was observed (Mann–Whitney U *P* = 0.59; Fig. 6A). Thus, it is likely that differences in cell size, and not cell number, mostly explain the difference in length between *C*. sp. 34 and *C. elegans*.

To quantify differences in cell size, the distances between homologous morphological landmarks in *C*. sp. 34 and *C. elegans* (*fog‐2*) adult females were measured (Fig. 6D–F). If the number of cells between these species is comparable, and their total length is different, then there should be differences in the distances between homologous landmarks. Indeed, the distances between homologous markers are greater in *C*. sp. 34 than *C. elegans* for every pair examined except for one (Fig. 6E). This is consistent with *C*. sp. 34 having larger cells than *C. elegans*. The one pair of morphological markers that are similarly spaced apart in *C*. *elegans* and *C*. sp. 34 is the posterior spermatheca and a posterior ventral cord neuronal nucleus (VD11; Fig. 6E). This similarity in length could be due to differences in gonad morphology, as this is influenced by the germline, which is often reduced in size or otherwise defective in *C*. sp. 34 (Fig. 4; Fig. S2; Fig. S3). In addition, the proportion of the total body size represented by the distance between two given homologous markers is largely comparable between species (Fig. 6F). This suggests that there is a global increase in cell size in *C*. sp. 34 compared to *C. elegans*.

In *C. elegans*, body size mutants typically reveal no changes in cell number despite changes in cell size (Suzuki *et al*. 1999; Flemming *et al*. 2000; Wang *et al*. 2002; Nagamatsu and Ohshima 2004; Soete *et al*. 2007). However, many such mutants (particularly those connected to TGF‐β signaling) have been shown to influence levels of somatic hypodermal endoreplication (Flemming *et al*. 2000; Morita *et al*. 2002; Nyström *et al*. 2002; Lozano *et al*. 2006; Fung *et al*. 2007), and levels of hypodermal ploidy are correlated with body size (Morita *et al*. 2002; Lozano *et al*. 2006). To address the possibility that variation in somatic nuclear ploidy may underlie the body size difference between *C. elegans* and *C*. sp. 34, we measured the DNA content of their hypodermal and intestinal nuclei using a densitometric approach (see methods). We found no differences in nuclear area (Fig. 6B) or somatic DNA content (Fig. 6C) for either cell type between *C. elegans* and *C*. sp. 34. Additionally, although no differences in total somatic nuclear number were observed, slight changes in hypodermal cell number have been associated with body size differences (Lozano *et al*. 2006). To investigate whether hypodermal cell number may be underlying body size differences, all of the hypodermal nuclei between the most anterior and posterior nuclei of the intestine were counted. No significant difference in hypodermal nucleus number between *C*. *elegans* and *C*. sp. 34 was observed (Fig. 6A). Thus the body size differences between *C. elegans* and *C*. sp. 34 may be promoted by mechanisms independent of hypodermal nucleus proliferation and endoreplication.

## Discussion

Genetic diversity drives phenotypic change. More than a hundred years of investigation has demonstrated that a multitude of quantitative traits can be readily transformed under natural and artificial selection (Castle 1911; Lewontin 1974; Kingsolver *et al*. 2001). Even substitutions of one or two simple genetic elements have been found to promote profound phenotypic changes within species (Martin and Orgogozo 2013). Thus, we would expect that a high degree of genetic diversity should provide ample material for the evolution of morphological diversity. The persistence of morphological stasis across long periods of time therefore remains an apparent paradox in evolutionary biology (Hansen and Houle 2004; Eldredge *et al*. 2005). Although often framed with respect to the fossil record, this observation also holds in extant taxa. Since the onset of the molecular era, the pace of descriptions of cryptic species has been exponential (Bickford *et al*. 2007), and the frequency of such species is not limited by phylum or geographical region (Pfenninger and Schwenk 2007). Morphological stasis in the face of genetic divergence is thus likely quite common and remains a largely ignored problem in evolutionary biology.

Such stasis is often explained by long‐term stabilizing selection, which purges divergent unfit forms from populations and reduces phenotypic variation (Charlesworth *et al*. 1982; Estes and Arnold 2007). Stabilizing selection is thought to be acting in most populations mainly because most organisms appear to be well‐adapted to their environments and thus some form of stabilizing selection must be ongoing (Parker and Smith 1990). However, others have argued that selection alone cannot explain the paradox of stasis (Williams 1992; Hansen and Houle 2004). One alternative explanation often invoked is the notion of developmental constraint (Smith *et al*. 1985). Here, phenotypic variation is limited by biases in the structure of the developmental genetic system itself, and divergence fails to occur because certain classes of phenotypes are not accessible to selection. This explanation is appealing due to the multitude of established such biases in developmental trajectories (Oster and Alberch 1982; Azhar *et al*. 2002; Braendle *et al*. 2010; Kavanagh *et al*. 2013); multiple examples of convergent phenotypic evolution promoted by the same nucleotide substitution, suggestive of limitations to the number of paths evolution can take (Gompel and Prud'homme 2009; Martin and Orgogozo 2013); and the prevalence of correlated traits, consistent with genetic constraints that influence the range of possible phenotypes (Hansen and Houle 2004; Futuyma 2010). Still others have suggested that the observation of long‐term stasis can be resolved by invoking an incomplete fossil record and the rapid turnover of locally adapted forms (Futuyma 1987; Williams 1992; Eldredge *et al*. 2005; Futuyma 2010), as well as the difficulty of empirically detecting acting stabilizing selection when populations are close to trait optima (Haller and Hendry 2014). Indeed, it is likely that a plurality of causes, including the joint action of selection and developmental constraint, contribute to patterns of long‐term morphological stasis (Hansen and Houle 2004; Futuyma 2010).

The nematode genus *Caenorhabditis* represents a striking example of phenotypic constancy in the face of genetic change. Despite roughly 20 million years of evolution (Cutter 2008), the 12 reported species of the *Elegans* group of *Caenorhabditis* are morphologically indistinguishable (Fig. 1D), and mating tests are often used delineate them from one another (Kiontke *et al*. 2011; Félix *et al*. 2014; Slos *et al*. 2017). This phenotypic constancy persists within the context of extreme genetic divergence within and between species. *C. elegans* and *C. briggsae* share about the same degree of genetic divergence as human and mouse (Kiontke *et al*. 2004). The male/female species *C. brenneri* and *C. remanei* harbor tremendous intraspecies polymorphism and are among the most genetically diverse metazoan species known (Dey *et al*. 2013), despite their cryptic species status (Sudhaus and Kiontke 2007). Furthermore, this morphological constancy has persisted despite ecological diversification in this group. Many *Caenorhabditis* species are generalists that are globally distributed and are found associated with a diverse group of invertebrate carriers (Kiontke and Sudhaus 2006). However, a number of other species in this group have a limited geographic range and form tight associations with specific insect vectors (Kiontke and Sudhaus 2006). It is remarkable that the divergent selective regimes associated with these different niches have resulted in such scant morphological change within this group.

*C. elegans* has a famously rigid pattern of development wherein the identity and fate of every cell from the fertilized embryo to the mature adult is known (Sulston *et al*. 1983). This set of cell divisions is unchanged across individuals and has allowed the genetic dissection of multiple developmental processes. Yet this developmental system is also highly conserved across multiple genetic backgrounds within species (Delattre and Félix 2001; Braendle and Félix 2009; Braendle *et al*. 2010) and even between species (Sternberg and Horvitz 1982; Delattre and Félix 2001; Zhao *et al*. 2008; Schulze and Schierenberg 2011). The highly conserved morphologies in this group are then promoted through highly conserved developmental processes. In tandem with the genetic and ecological diversity observed across the *Caenorhabditis* genus, this is all suggestive of a prevailing role for developmental constraint along its millions of years of evolution.

*C*. sp. 34 clearly bucks this overall pattern, as it displays a morphology and ecology that are distinct departures from its close relatives. Thus developmental constraint alone cannot be driving the patterns of stasis observed in this group. Here, we examined the broad developmental patterns of this divergent species. Together with the extensive background knowledge of the *C. elegans* model system, the roles of constraint and selection in maintaining the general pattern of phenotypic constancy in this group can be interrogated. The existence of mutations in every known protein‐coding gene in *C. elegans* (Thompson *et al*. 2013) provides a window into the universe of evolutionarily‐accessible phenotypes that can potentially describe the extent of developmental constraint in this system.

Mutations that affect the body size were among the first described in *C. elegans* (Brenner 1974), and genes that when defective promote long (lon), small (sma), and dumpy (dpy; that is, small and fat) phenotypes are among the most notable in this system. Thus, the existence of a novel species that is long (that is, *C*. sp. 34) does not in itself reveal a new region of phenotypic space that was thought to be inaccessible or constrained. However, the developmental biology of these mutants, and their similarity to *C*. sp. 34, reveals insights into the limits of evolutionary trajectories in this group. For instance, given the size difference, it is remarkable that no detectable difference in total somatic cell number (nor hypodermal cell number) between *C. elegans* and *C*. sp. 34 was found (Fig. 6A). Similarly, changes in cell number typically have not been detected in *C. elegans* body size mutants (Suzuki *et al*. 1999; Flemming *et al*. 2000; Wang *et al*. 2002; Nagamatsu and Ohshima 2004; Soete *et al*. 2007). This is also consistent with the general observation that nucleus number alone is a poor predictor of body size in rhabditid nematodes (Flemming *et al*. 2000). However, there is an interaction between cell number and hypodermal ploidy that is predictive of body size (Flemming *et al*. 2000). In *C. elegans*, many genes known to regulate body size are components of or otherwise interact with the TGF‐β signaling pathway (Gumienny and Savage‐Dunn 2013), and mutations in a number of these genes have also been shown to have correlated changes in hypodermal ploidy (Flemming *et al*. 2000; Morita *et al*. 2002; Nyström *et al*. 2002; Lozano *et al*. 2006; Fung *et al*. 2007). Here, densitometric image analysis of DNA‐stained animals revealed no differences in hypodermal DNA content between *C. elegans* and *C*. sp. 34 (Fig. 6C), suggesting that hypodermal endoreplication may not be the main driver of body size differences between these species. Furthermore, although there are genes that influence cell lineage (and subsequently cell number (Horvitz and Sulston 1980)), and genes that control germ line proliferation (Francis *et al*. 1995), there are, to the best of our knowledge, no mutants with increased body size due to increased cell number in *C. elegans*. Thus, the evolution of body size in *Caenorhabditis* is likely restricted to paths that increase cell size as opposed to cell number.

In addition, *C. elegans* body size mutants typically only reveal their differences from wild‐type after embryogenesis (Suzuki *et al*. 1999; Morita *et al*. 2002; Nyström *et al*. 2002; Hirose *et al*. 2003; Soete *et al*. 2007). Likewise, we find that *C*. sp. 34 adults are on average 64% longer than *C*. *elegans* adults but that their embryos are only 19% longer (Fig. 5). Thus, both *C. elegans* body size mutants and *C*. sp. 34 largely reveal their differences postembryonically, which may belie another constraint evolution must operate under to change body size in this group. In the same vein, most of the known long mutants in *C. elegans* do not reveal apparent differences in width (Brenner 1974; Soete *et al*. 2007) (although *egl‐4* mutants are exceptionally gigantic (Hirose *et al*. 2003)), the same of which can be said for *C*. sp. 34 and *C. elegans* (Fig. 5K). And finally, to the best of our knowledge, there are no mutants in *C. elegans* that modulate body size by increasing the size of one tissue relative to the others; *C*. sp. 34 likewise reveals a global increase in length (Fig. 6E and F). Thus, when framed within the context of the extensive literature of the *C. elegans* model system, the broad developmental patterns of its morphologically divergent close relative do in fact reveal some developmental biases have likely helped to shape the specific pattern and/or mechanism of this divergence.

But what proximal, physical mechanisms might be driving the evolution of cell size in *C*. sp. 34? A number of explanations (which are not mutually exclusive) for the basis of body size regulation in *C. elegans* have been advanced (Tuck 2014): nutrition‐related signaling (Bishop and Guarente 2007; Tain *et al*. 2008); germ line signaling (Patel *et al*. 2002); cuticle structure (Johnstone 2000; Nyström *et al*. 2002; Suzuki *et al*. 2002; Soete *et al*. 2007; Roberts *et al*. 2010; Schultz *et al*. 2014); hypodermal cytoskeletal organization (Praitis *et al*. 2005); global changes in gene expression and protein synthesis (Nagamatsu and Ohshima 2004; Roberts *et al*. 2010); and changes in DNA content, whether via germ line ploidy (Lozano *et al*. 2006) or endoreplication in the hypodermis (Flemming *et al*. 2000; Morita *et al*. 2002; Nyström *et al*. 2002; Lozano *et al*. 2006; Fung *et al*. 2007; Tain *et al*. 2008). An additional, seemingly neglected physical explanation for body size change is cell stacking, which was noted in observations of *C. elegans* body size mutants that nonetheless showed no differences in cell number nor cell volume (Knight *et al*. 2002). *C*. sp. 34 may also be larger due to expansion of extracellular space. Furthermore, changes in the timing of the cell cycle are known to covary with cell size in eukaryotes (Nurse 1975; Stocker and Hafen 2000), and this mechanism could possibly account for both the changes in *C*. sp. 34 body size and developmental rate. The examination of oocytes arrested in prophase revealed no differences in germ line ploidy between *C. elegans* and *C*. sp. 34 (Fig. 4), and densitometric image analysis of DNA‐stained animals revealed no differences in hypodermal DNA content (Fig. 6F). Mutations in a number of TGF‐β signaling pathway genes have been shown to have correlated changes in hypodermal ploidy (Flemming *et al*. 2000; Morita *et al*. 2002; Nyström *et al*. 2002; Lozano *et al*. 2006; Fung *et al*. 2007; Tain *et al*. 2008) (although one study was unable detect an effect of TGF‐β signaling on hypodermal ploidy (Nagamatsu and Ohshima 2004)). Additionally, experiments with drugs that inhibit DNA synthesis also suggest that TGF‐β signaling promotes body size mostly through hypodermal endoreplication (Lozano *et al*. 2006). However, many genes that regulate body size but are not clearly connected to TGF‐β signaling do not appear to impact hypodermal ploidy (Nyström *et al*. 2002; Nagamatsu and Ohshima 2004; Fung *et al*. 2007; Soete *et al*. 2007; Chen *et al*. 2008). Taken together, this would suggest the speculative interpretation that *C*. sp. 34 might be longer than *C. elegans* because of genetic changes in pathways that are parallel to TGF‐β signaling. In any case, as body size is a typically highly complex trait and that there are multiple candidate genes and pathways that regulate body size in *C. elegans*, understanding the mechanisms underlying body length change in *C*. sp. 34 is likely to be a fruitful area of research in the coming years.

And although *C*. sp. 34 appears to be operating under constraints generated by known developmental processes, its tremendous departure in form from its close relatives remains to be accounted for. As mentioned above, *Caenorhabditis* species do display diversity in geographic range and phoretic‐carrier association. However, it appears that a major aspect of their ecological niche is shared among species in this group: *Caenorhabditis* nematodes generally proliferate on rotting plant material (Kiontke *et al*. 2011). And although there is almost no variation in somatic cell lineage and cell number (Delattre and Félix 2001; Zhao *et al*. 2008; Braendle and Félix 2009; Braendle *et al*. 2010), there is ample variation in body size in *Caenorhabditis* (Hodgkin and Doniach 1997; Knight *et al*. 2001; Snoek *et al*. 2014) for selection to act upon (Azevedo *et al*. 2002), despite the general constancy in length in this group (Fig. 1D). Thus, in rotting‐plant *Caenorhabditis*, body length variation is likely held in check by stabilizing selection, while the cell lineage seems to be a developmental constraint across the genus. In contrast, *C*. sp. 34 proliferates in the fresh figs of *Ficus septica*, the microcosm of which is very different from that of rotting fruit, harboring a unique suite of specific wasps, nematodes, and other microorganisms (Herre *et al*. 2008). This major ecological shift is nearly certain to coincide with a major shift in selective regimes, allowing for the opportunity of novel morphological change in the case of *C*. sp. 34. Thus, the common ecological niches of most *Caenorhabditis* species allow stabilizing selection to maintain a morphology and body length that is suited for rotting‐plant bacteriophagy. But, the move to a totally novel niche, as in the case of *C*. sp. 34, has allowed divergent selection to promote novel morphologies within the constraints imposed by its developmental system (i.e., the mostly invariant cell lineage). Thus selection and constraint act jointly to promote the pattern of morphologies observed in *Caenorhabditis*. In this way, the comparative development approach, together with the context of model systems genetics, can inform long‐standing evolutionary questions regarding the interplay of selection and developmental constraint over geological timescales.

## DATA ACCESSIBILITY

Data not included in the supplemental material are available from the authors upon request.

Associate Editor: A. Goswami

## Supporting information


**Figure S1**. Localities of sequenced C. sp. 34 animals.
**Figure S2**. Germ line abnormalities in *C*. sp. 34.
**Figure S3**. *C*. sp. 34 gonads are smaller than *C*. elegans gonads.
