# Supplementary material for: Dramatic evolution of body length due to postembryonic changes in cell size in a newly discovered close relative of Caenorhabditis elegans
Source: Evol Lett. 2018 Jul 16;2(4):427–41. doi: 10.1002/evl3.67 (PMC6121821; doi:10.1002/evl3.67)
Supplement: Supplementary file 1 — Figure S1. Localities of sequenced C. sp. 34 animals. Figure S2. Germ line abnormalities in C. sp. 34. Figure S3. C. sp. 34 gonads are smaller than C. elegans gonads. [file EVL3-2-427-s001.pdf]

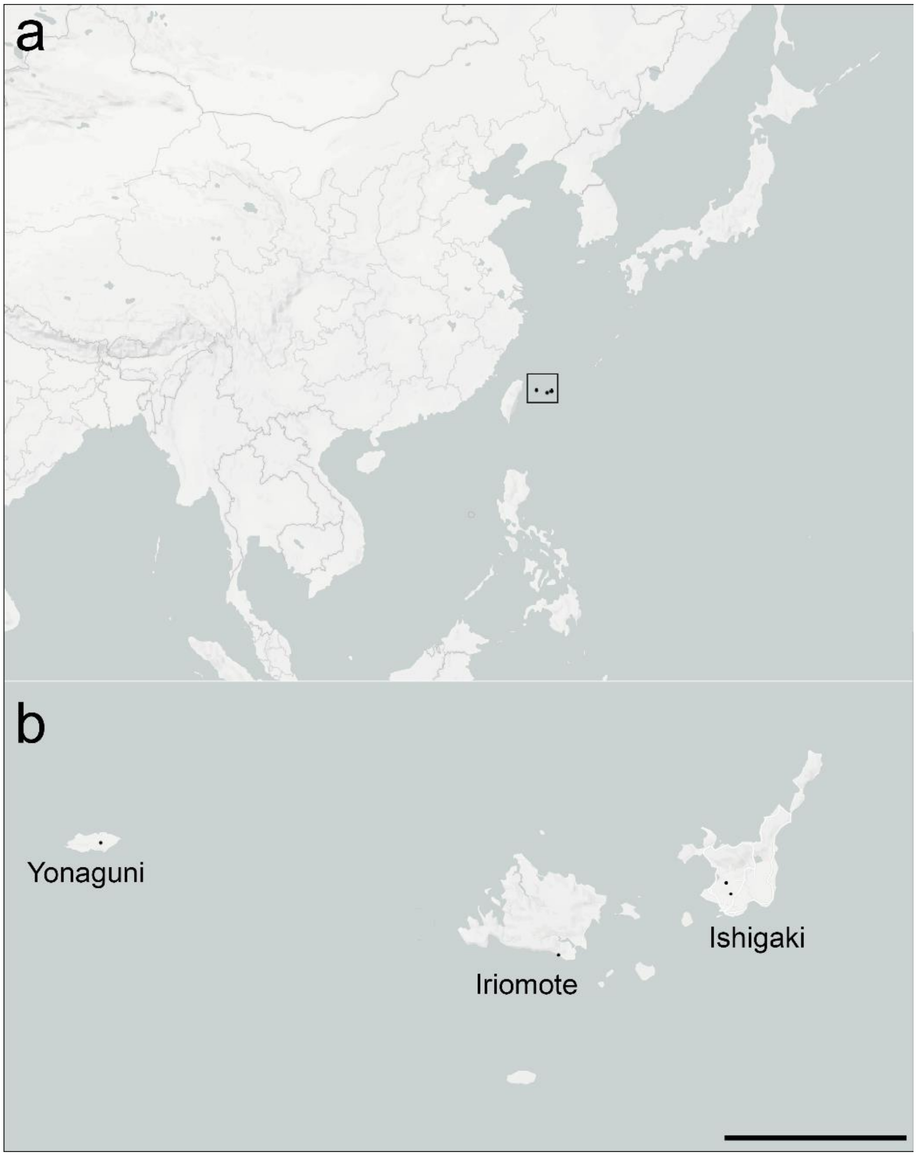

Figure S1. Localities of sequenced *C.* sp. 34 animals. (a) *C.* sp. 34 was discovered in association with *F. septica* on the Sakishima islands of Okinawa, Japan (box). (b) In 2016, 21 individual nematodes were harvested from figs from Ishigaki, Iriomote, and Yonaguni island. These individuals were utilized for sequencing. The scale bar represents 30 km. See Supplemental Table S1 for the GPS coordinates of these isolates.

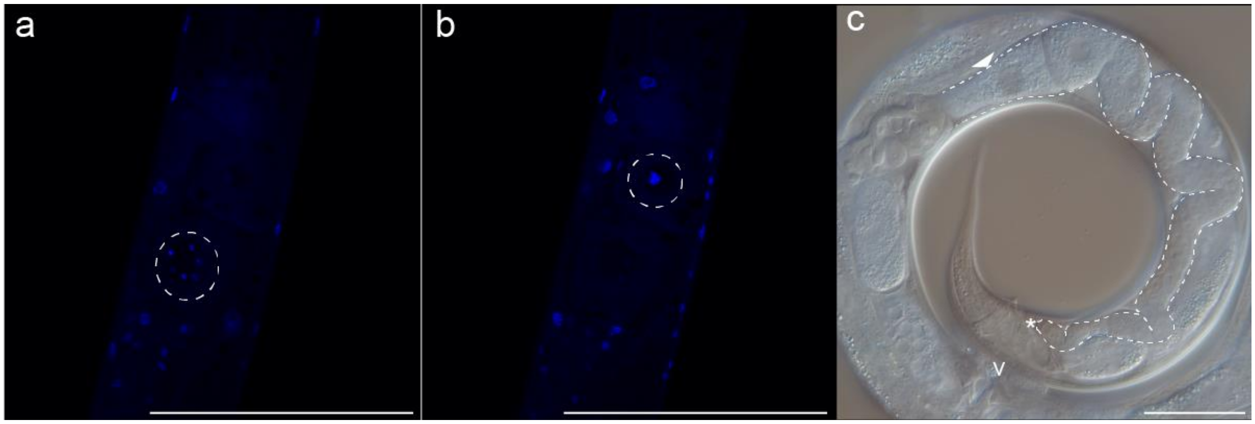

Figure S2. Germ line abnormalities in *C.* sp. 34. (a-b) Differentiated oocytes can display chromosomal defects. (a) The six chromosomes of a wild-type oocyte nucleus are circled. (b) In the adjacent distal oocyte of the same animal (different focal plane), the chromosomes cannot be delineated and the DNA is clustered in the center of the nucleus (circled). (c) Gonads can display unusual pathfinding phenotypes. Typically, the *Caenorhabditis* gonad has one bend and is U-shaped. Here, the gonad (outlined) reveals multiple bends and a distal tip cell in the posterior end of the animal, indicative of irregular cell migration. Arrowhead, most proximal differentiated oocyte; Asterisk, distal end of the gonad; v, vulva. Scale bars are 100 microns in all panels.

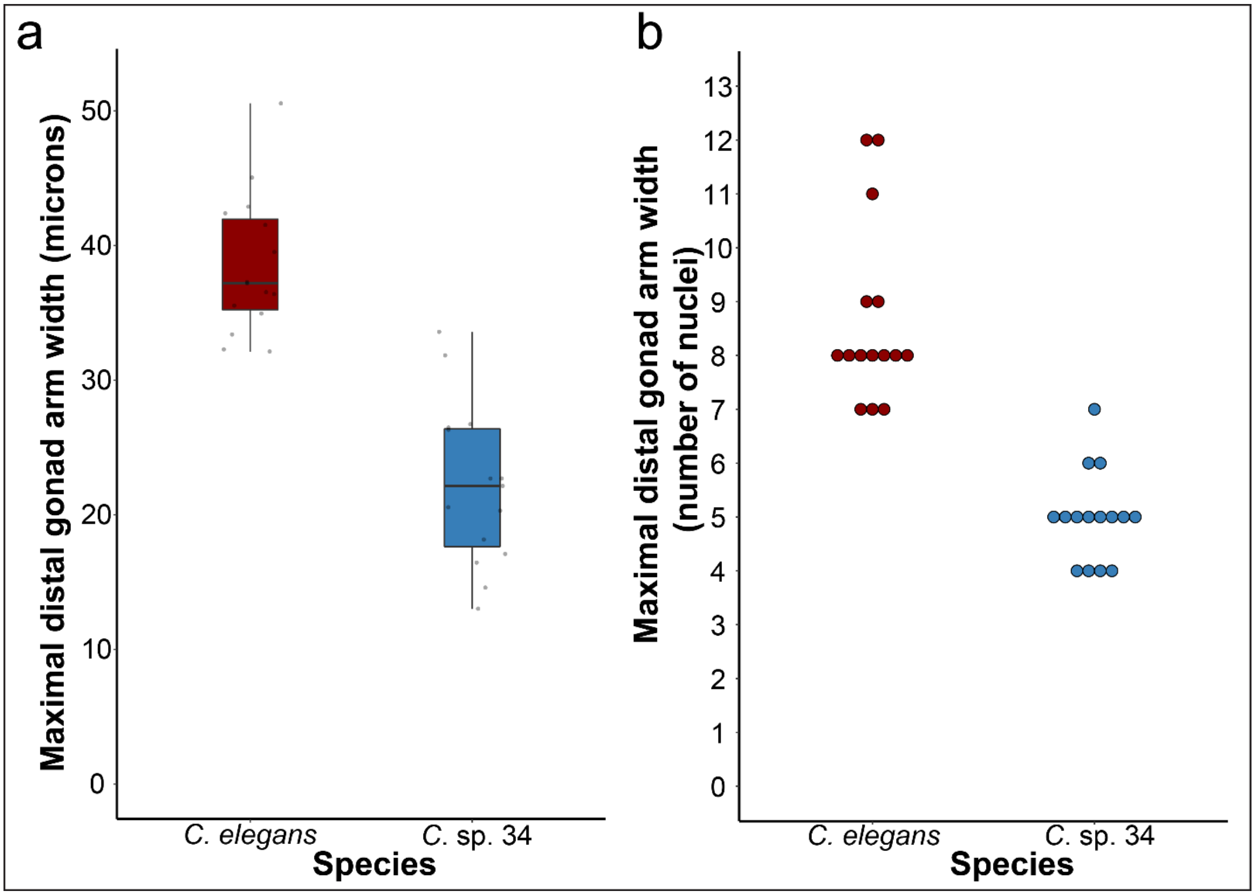

Figure S3. *C*. sp. 34 gonads are smaller than *C. elegans* gonads. (a) The maximal hermaphrodite/female distal gonad arm width measured in microns. *C. elegans* median = 37 microns, range = 32-51 microns; *C*. sp. 34 median = 22 microns, range = 13-34 microns. Mann-Whitney U p < 0.0001. N=15 worms for both species. (b) The maximal hermaphrodite/female distal gonad arm width measured in nucleus number. That is, the number of germ cell nuclei spanning the maximal width of the distal gonad arm. Same data as in panel (a). *C. elegans* median = 8 nuclei, range = 7-12 nuclei; *C*. sp. 34 median = 5 nuclei, range = 4-7 nuclei. Mann-Whitney U p < 0.0001.

Document S1: Discussion of a phylogenetic analysis

In this study, one maximum likelihood phylogenetic analysis of 287 concatenated partial protein sequences across 24 *Caenorhabditis* taxa places *C.* sp. 34 solidly within the genus and incidentally the closest reported relative of *C. elegans*. *C.* sp. 34 is clearly a close relative of *C. elegans*. But how much confidence can be placed in the hypothesis suggested by this particular phylogeny?

One approach is to compare this topology with previously reported phylogenetic analyses. This phylogeny is largely concordant with the trees reported in Kiontke et al. 2011 and Slos et al. 2017, but there are notable differences.

C. virilis is reported as being a member of the "*Drosophilae* supergroup" (Kiontke et al. Fig. 1) in both Slos et al. and Kiontke et al. Here, it has been placed as ancestral to the *Drosophilae* supergroup with low bootstrap support. Here, *C. brenneri* is placed as sister to *C. doughteryi* (a relationship reported in Kiontke et al.), but in Slos et al., *C. brenneri* is reported as ancestral to a *C. doughertyi*-*C. wallacei*-*C. tropicalis* clade. Additionally, this study placed *C.* sp. 40 as the sister species to *C.* sp. 26, whereas Slos et al. put *C. sinica* as the closest relative of *C.* sp. 40. Here, the clade including *C. japonica*, *C. afra*, and *C. nouraguensis* (the "*Japonica* group;" Kiontke et al. 2011 Fig. 1) was recovered with low support and a topology different from previous studies. Both Slos et al. and Kiontke et al. report a *Japonica* group clade with high support and with *C. japonica* as the most ancestral lineage; in this tree *C. nouraguensis* is the more ancestral lineage. This study utilized only four *Japonica* group members (as defined by the previous studies): *C. japonica*, *C. afra*, *C. nouraguensis*, and *C.* sp. 32. Slos et al. used five *Japonica* group members, and Kiontke et al. used six. So, it is possible that a lack of taxa contributed to the inability to resolve this particular group with high support in this analysis. Aside from these differences, this tree is largely consistent with those previously reported. The general agreement of this analysis with previous studies lends some confidence to the inferred *C. elegans*-*C.* sp. 34 clade.

Bootstrap replicates are also used to ascertain confidence in inferred topologies. This analysis reported seven nodes without 100% bootstrap support. Two nodes are related to the *Japonica* group, which includes branches with low support in previous studies (Kiontke et al. 2011; Slos et al. 2017). One of these is the node leading to *C. virilis* and descendants, as described above. Another is the node leading to *C.* sp. 21 and its descendants. Another is the node leading the *C. doughertyi*-*C. brenneri* lineage, which also has tended to have lower support (Kiontke et al. 2011) or has not been reported (Slos et al. 2017). Another is the node leading to the *C.* sp. 26-*C.* sp. 40 clade (discussed above), and the last is the node supporting the *C.* sp. 34-*C. elegans* clade. The seven bootstrap replicate topologies that did not have this clade are shown (Figure). Six place *C. elegans* as ancestral to the rest of the clade, and one places *C.* sp. 34 as the outgroup to the rest of the clade.

Phylogenetic inference is influenced by the number of taxa and loci included in the analysis, and the management of missing data has long been an unresolved issue in phylogenetics (Weins et al. 2006; Nabhan and Sarkar 2011). The analysis reported here did not use a genome-wide set of loci and only used less than half of the known *Caenorhabditis* species (Ferarri et al. 2017). Thus, it is likely that missing data is influencing the analysis, and more taxa or loci are probably needed to resolve this relationship.

However, even if more data can resolve a single topology for the species tree, it is clear that the underlying gene trees often have conflicting topologies and therefore variant

evolutionary histories (Degnan and Rosenburg 2009). Biologically-relevant phylogenetic discordance can be caused by incomplete lineage sorting, ancient introgression, and horizontal gene transfer (Hahn and Nakleh 2016). Further, such patterns of discordance can vary with patterns of genomic structure, providing invaluable biological information that is lost with more traditional concatenation approaches (Martin et al. 2013; Fontaine et al. 2015). As the number of *Caenorhabditis* species and genome sequences has rapidly expanded in recent years (Slos et al. 2017), it is clear that a genus-wide, genome-wide phylogenetic approach that more accurately captures the complexity of *Caenorhabditis* history will soon be undertaken.

References

Degnan, J. H., & Rosenberg, N. A. (2009). Gene tree discordance, phylogenetic inference and the multispecies coalescent. *Trends in ecology & evolution*, *24*(6), 332-340.

Ferrari, C., Salle, R., Callemeyn-Torre, N., Jovelin, R., Cutter, A. D., & Braendle, C. (2017). Ephemeral-Habitat Colonization And Neotropical Species Richness Of *Caenorhabditis* Nematodes. *bioRxiv*, 142190.

Fontaine, M.C., Pease, J.B., Steele, A., Waterhouse, R.M., Neafsey, D.E., Sharakhov, I.V., Jiang, X., Hall, A.B., Catteruccia, F., Kakani, E. and Mitchell, S.N., 2015. Extensive introgression in a malaria vector species complex revealed by phylogenomics. *Science*, *347*(6217), p.1258524.

Hahn, M. W., & Nakhleh, L. (2016). Irrational exuberance for resolved species trees. *Evolution*, *70*(1), 7-17.

Kiontke, K. C., Félix, M. A., Ailion, M., Rockman, M. V., Braendle, C., Pénigault, J. B., & Fitch, D. H. (2011). A phylogeny and molecular barcodes for *Caenorhabditis*, with numerous new species from rotting fruits. *BMC evolutionary biology*, *11*(1), 339.

Martin, S.H., Dasmahapatra, K.K., Nadeau, N.J., Salazar, C., Walters, J.R., Simpson, F., Blaxter, M., Manica, A., Mallet, J. and Jiggins, C.D., 2013. Genome-wide evidence for speciation with gene flow in Heliconius butterflies. *Genome Research*, *23*(11), pp.1817-1828.

Nabhan, A. R., & Sarkar, I. N. (2011). The impact of taxon sampling on phylogenetic inference: a review of two decades of controversy. *Briefings in Bioinformatics*, *13*(1), 122-134.

Slos, D., Sudhaus, W., Stevens, L., Bert, W., & Blaxter, M. (2017). *Caenorhabditis monodelphis* sp. n.: defining the stem morphology and genomics of the genus *Caenorhabditis*. *BMC Zoology*, *2*(1), 4.

Wiens, J. J. (2006). Missing data and the design of phylogenetic analyses. *Journal of biomedical informatics*, *39*(1), 34-42.

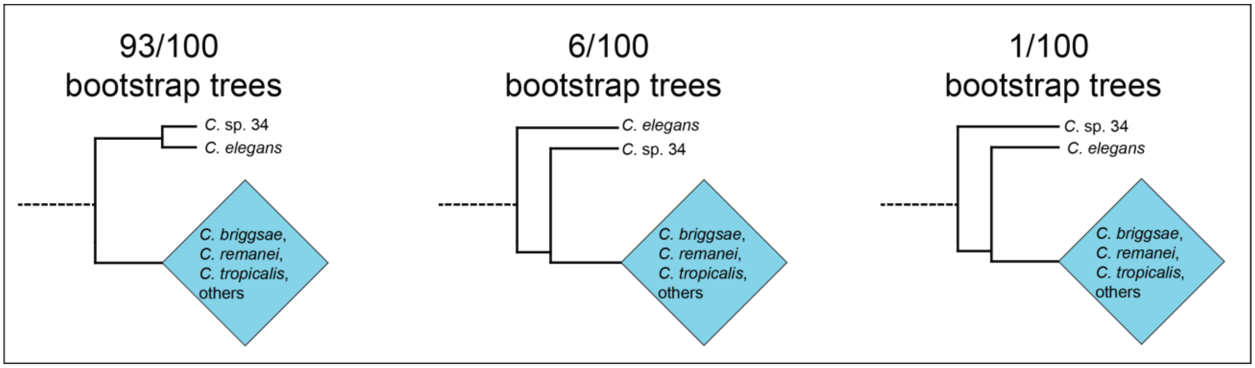

Figure. *C. elegans*-*C.* sp. 34 relationships among 100 bootstrap replicate trees.

Document S2: Mating tests between *C*. sp. 34 and *C. elegans*

Mating tests methods

To determine the extent of reproductive isolation between *C*. sp. 34 and *C. elegans*, mating tests between presumed species were performed. To ensure virginity of females, animals were picked as immature L4 larvae, and isolated from males for one (*C. elegans*) or two (*C*. sp. 34) days before mating. In most crosses, the *C. elegans fog-2 (q71)* mutation was used to avoid confusing self-progeny with cross-progeny. In some crosses, males from the *C. elegans* strain QG2288 were used because of their vigorous mating behavior (Noble et al. 2015). For *C*. sp. 34, the wild isolate strain NKZ1 was used for all crosses. For a subset of *C. elegans* ⚥ x *C*. sp. 34 ♂ crosses, *C. elegans* QG2288 hermaphrodites purged of self-sperm were used. Here, hermaphrodites were monitored and moved to new plates daily until they stop producing self-progeny. Then, they were used for mating tests. The ability of *C*. sp. 34 males to sterilize *C. elegans* hermaphrodites (Ting et al. 2014) was gauged with wild-type *C. elegans* QG2288. Conspecific crosses were monitored until viable F_2 progeny were observed. Interspecific crosses were monitored daily for three days after the cross was initiated for signs of successful copulation. These included the presence of F_1 embryos, the deposition of copulatory plugs, and the presence of male mating behavior. A fraction of females used in these crosses were also monitored for successful sperm transfer under Nomarski microscopy. All crosses were performed at 25°C.

Three cross designs were utilized for mating tests. Initial crosses were performed with three females and five males. However, as no interspecies embryo production was observed in these, other interspecies cross designs were implemented. Crosses using eight males and three females were performed. In addition, two reciprocal, interspecific large crosses with forty males and thirty females were performed. Sample sizes for various crosses are shown in Table 2.

Results and Discussion

In *Caenorhabditis*, the preponderance of cryptic species has led to the utilization of hybridization as an aspect of species delimitation (Felix et al. 2014). Additionally, the promise of a new close relative of *C. elegans* opens up the possibility of using a classic model system to investigate reproductive isolation, the evolution of hermaphroditism, and the evolution of body size and developmental timing (among other phenotypes) with genetic trait mapping approaches. To this end, such mating tests were performed between *C*. sp. 34 and *C. elegans*. For some crosses with *C. elegans*, a *fog-2* mutation (wherein hermaphrodites do not produce sperm and males are wild-type (Schedl and Kimble 1988)) was used to prevent the generation of self-progeny. Crosses were also performed with *C. elegans* strain QG2288, known to mate more vigorously than N2 (Noble et al. 2015). Crosses were assayed for the presence of mating behavior, copulatory plug deposition, embryo production, and sperm transfer (which was performed via Nomarski microscopy).

The results of the mating tests are summarized below (Table 1). No interspecies crosses produced viable progeny, whereas all *C. elegans* (n = 10) and *C*. sp. 34 (n = 9) conspecific crosses produced abundant F_1 and F_2 adults. *C. elegans* males were observed mating with *C*. sp. 34 females (Figure 1a), whereas *C*. sp. 34 males were rarely observed mating with *C. elegans fog*-2 pseudo-females. None of these crosses resulted in embryo production. Likewise, no hybrid embryos were observed when wild-type, aged *C. elegans* hermaphrodites (who had ceased laying

self-embryos) were crossed with *C*. sp. 34 males. *C*. *elegans* males did not inseminate *C*. sp. 34 females (n = 16 females observed under DIC microscopy after test cross). *C*. sp. 34 males did not deposit copulatory plugs and transfer sperm to *C*. *elegans* pseudo-females. Consistent with this, *C*. sp. 34 males were unable to sterilize young, wild-type *C*. *elegans* hermaphrodites (n = 9 hermaphrodites in three crosses), as has been observed in other interspecific crosses of *Caenorhabditis* (Ting et al. 2014).

Despite their obvious phenotypic distinctiveness, mating tests indeed confirm that *C.* sp. 34 and *C. elegans* are good biological species (Table 1). Isolating barriers can occur at many steps in reproduction (Coyne and Orr 2004), and in *Caenorhabditis*, reproductive isolation usually occurs post-zygotically as most interspecific matings produce inviable F_1 embryos (Baird and Seibert 2013). Viable and fertile hybrids occur in some species pairs (Woodruff et al. 2010; Kolowzka et al. 2012; Kiontke et al. 2011), and a bizarre form of gametic isolation via ectopic sperm localization has also been observed (Ting et al. 2014).

Here, however, all crosses revealed no evidence of embryo production or sperm transfer (Table 1). Mating behavior was observed when crossing *C. elegans* males with *C.* sp. 34 females, but mating behavior was rarely observed in the reciprocal cross (Table 1). In no interspecific cross was successful sperm transfer observed (Table 1). Not all interspecific crosses in the Elegans group produce hybrid embryos, but all involve copulation behavior and most involve sperm transfer (Baird and Seibert 2013; Hill and L'Hernault 2002). In one report, *C. elegans* males were not able to inseminate *C. remanei* females, despite successful insemination in the reciprocal cross (Hill and L'Hernault 2002). *C. elegans* and *C.* sp. 34 are then isolated by a reproductive barrier that is rare in the Elegans group. It remains unclear the degree to which this barrier is mechanical and/or behavioral. Mechanical isolation refers to an inhibition of successful copulation due to incompatibilities of reproductive structures, entailing classic "lock and key" barriers to reproduction (Coyne and Orr 2004). It is possible that the size differences between species may lead to an inability of the male tail to properly locate the interspecific female vulva. Or, given possible differences in mail tale structure between fig-associated species and *C. elegans*, it is possible that interspecific spicule insertion is difficult and that a true "lock and key" barrier is in effect. Further analysis of *C.* sp. 34 male morphology and interspecific mating behavior is needed to delineate this possibility. If true, this is quite notable because despite its discussion in the literature and textbooks, actual case studies of such barriers are limited (Coyne and Orr 2004).

References

Baird, S.E. & Seibert, S.R. (2013). Reproductive isolation in the Elegans-Group of Caenorhabditis. *Natural Science*. 5(4), e30825.

Coyne, J. A., & Orr, H. A. (2004). *Speciation*. Sunderland, MA: Sinauer Associates.

Félix, M. A., Braendle, C., & Cutter, A. D. (2014). A streamlined system for species diagnosis in Caenorhabditis (Nematoda: Rhabditidae) with name designations for 15 distinct biological species. *PLoS One*, *9*(4), e94723.

Hill, K. L., & L'Hernault, S. W. (2001). Analyses of reproductive interactions that occur after heterospecific matings within the genus Caenorhabditis. *Developmental biology*, *232*(1), 105-114.

Kiontke, K. C., Félix, M. A., Ailion, M., Rockman, M. V., Braendle, C., Pénigault, J. B., & Fitch, D. H. (2011). A phylogeny and molecular barcodes for Caenorhabditis, with numerous new species from rotting fruits. *BMC Evolutionary Biology*, *11*(1), 339.

Kozlowska, J. L., Ahmad, A. R., Jahesh, E., & Cutter, A. D. (2012). Genetic variation for postzygotic reproductive isolation between Caenorhabditis briggsae and Caenorhabditis sp. 9. *Evolution*, *66*(4), 1180-1195.

Noble, Luke M., et al. "Natural variation in plep-1 causes male-male copulatory behavior in C. elegans." *Current Biology* 25.20 (2015): 2730-2737.

Schedl, T., & Kimble, J. (1988). fog-2, a germ-line-specific sex determination gene required for hermaphrodite spermatogenesis in Caenorhabditis elegans. *Genetics*, *119*(1), 43-61.

Ting, J. J., Woodruff, G. C., Leung, G., Shin, N. R., Cutter, A. D., & Haag, E. S. (2014). Intense sperm-mediated sexual conflict promotes reproductive isolation in Caenorhabditis nematodes. *PLoS Biol*, *12*(7), e1001915.

Woodruff, G. C., Eke, O., Baird, S. E., Félix, M. A., & Haag, E. S. (2010). Insights into species divergence and the evolution of hermaphroditism from fertile interspecies hybrids of Caenorhabditis nematodes. *Genetics*, *186*(3), 997-1012.

Cross	Result	N*
C. elegans fog-2(q71) **conspecific**	Viable F_1 and F_2 progeny	3
C. **sp. 34 conspecific**	Viable F_1 and F_2 progeny	9
C. **sp. 34 ♀ X *C. elegans* QG2288 ♂**	Mating behavior observed, no F_1 embryos observed	10
C. **sp. 34 ♀ X *C. elegans fog-2* ♂**	Mating behavior sometimes observed, no F_1 embryos, no sperm transfer observed (n=16 females)	1
C. elegans fog-2 **pseudo-♀ X *C.* sp. 34 ♂**	No mating behavior observed, no F_1 embryos, no sperm transfer observed (n=15 pseudo-females)	8
C. elegans **QG2288 old† ⚥ X *C.* sp. 34 ♂**	No mating behavior observed, no F_1 embryos observed	10
C. elegans **QG2288 ⚥ X *C.* sp. 34 ♂**	Mating behavior observed, no sterilization of hermaphrodites observed.	3

Table 1. *C.* sp. 34 is a distinct biological species. See methods for details. The wild isolate strain of *C.* sp 34, NKZ1, was used for all crosses. Briefly, various cross designs were employed, and crosses were monitored for three days for evidence of successful mating, or until the presence of viable F_2 in the case of conspecific crosses. A subset of females was observed under Nomarski optics for evidence of sperm transfer. *N=number of crosses performed, breakdown by cross design in Table 2. †= hermaphrodites with self-sperm depleted by aging.

Cross	3 ♀ x 5 ♂	3 ♀ x 8 ♂	30 ♀ x 40 ♂	Total
***C. elegans fog-2(q71)* conspecific**	3	-	-	3
***C.* sp. 34 conspecific**	9	-	-	9
***C.* sp. 34 ♀ X *C. elegans* QG2288 ♂**	10	-	-	10
***C.* sp. 34 ♀ X *C. elegans fog-2* ♂**	-	-	1	1
***C. elegans fog-2* pseudo-♀ X *C.* sp. 34 ♂**	5	2	1	8
***C. elegans* QG2288 old ⚥* X *C.* sp. 34 ♂**	10	-	-	10
***C. elegans* QG2288 ⚥ X *C.* sp. 34 ♂**	3	-	-	3

Table 2. Sample sizes of various interspecies crosses. *Old hermaphrodites are wild-type animals that have utilized all of their self-sperm before the start of the cross. “-“, no crosses of this type were made.
